# Moving towards accurate and early prediction of language delay with network science and machine learning approaches

**DOI:** 10.1038/s41598-021-85982-0

**Published:** 2021-04-14

**Authors:** Arielle Borovsky, Donna Thal, Laurence B. Leonard

**Affiliations:** 1 Department of Speech, Language, and Hearing Sciences, Purdue University, West Lafayette, IN 47906 USA; 2 School of Speech, Language, and Hearing Sciences, San Diego State University, San Diego, CA USA

**Keywords:** Psychology, Human behaviour, Risk factors, Machine learning

## Abstract

Due to wide variability of typical language development, it has been historically difficult to distinguish typical and delayed trajectories of early language growth. Improving our understanding of factors that signal language disorder and delay has the potential to improve the lives of the millions with developmental language disorder (DLD). We develop predictive models of low language (LL) outcomes by analyzing parental report measures of early language skill using machine learning and network science approaches. We harmonized two longitudinal datasets including demographic and standardized measures of early language skills (the MacArthur-Bates Communicative Developmental Inventories; MBCDI) as well as a later measure of LL. MBCDI data was used to calculate several graph-theoretic measures of lexico-semantic structure in toddlers’ expressive vocabularies. We use machine-learning techniques to construct predictive models with these datasets to identify toddlers who will have later LL outcomes at preschool and school-age. This approach yielded robust and reliable predictions of later LL outcome with classification accuracies in single datasets exceeding 90%. Generalization performance between different datasets was modest due to differences in outcome ages and diagnostic measures. Grammatical and lexico-semantic measures ranked highly in predictive classification, highlighting promising avenues for early screening and delineating the roots of language disorders.

## Introduction

Developmental language disorder (DLD) is a common language learning impairment that affects approximately 7% of school-aged children^1^. This disorder is characterized by deficits in aspects of language functioning, while showing normal learning/cognitive capacity, and the absence of biomedical explanations for a learning delay, such as brain injury or hearing impairment^2^. DLD is associated with elevated risk for a host of negative psycho-social and health outcomes including: psychiatric disorders^3^; incarceration and juvenile delinquency^4^**;** experiencing sexual assault^5^; lowered educational attainment and occupational status^6^; social and interpersonal difficulties^7^; and elevated healthcare costs^8^.

DLD is rarely diagnosed before a child approaches school age. Nevertheless, the roots of this disorder are present even from infancy: Parents of children with DLD often report in retrospect that their child experienced delays in meeting early communication milestones^9^. For example, Rudolph and Leonard (2016) asked parents of 150 school-aged children (76 with DLD) to recall when their child reached two early language milestones (onset of talking and onset of multi-word utterances). About half (55%) of children with DLD were reported (in retrospect) to exhibit early delays in onset of talking (i.e. saying no words by 15 months), and/or onset of multi-word combinations (i.e. not producing multi-word utterances by 24 months), whereas only 14% of children with typical development (TD) exhibited a delay in one of these early milestones. Numerous groups have sought to evaluate whether standardized parental report measures of language can prospectively identify school-age language disorders from infancy and toddlerhood^10^. While standardized measures have identified broad differences between groups who do and do not eventually experience DLD, no early measure to date has had sufficient sensitivity or specificity to serve as a reliable clinical marker of later disorder.

Using powerful computational tools, we seek to improve early DLD risk assessment via parental report of early communication skills. Specifically, we focus on theory-driven measures that use as their starting point data derived from the MacArthur-Bates Communicative Development Inventory (MBCDI). The MBCDI has many desirable properties that encourage prospects for wide distribution for screening in early clinical and educational settings. It is a reliable, validated, and norm-referenced caregiver inventory of expressive vocabulary and other early language skills that is easy to administer in a variety of settings; and it does not require a professional specialist for scoring^11^. The MBCDI Words and Sentences form assesses language skills in 16- to 30-month-olds, and associates with numerous concurrent language processing abilities^12,13^. However, attempts to predict later language diagnostic outcomes from this measure with standard linear modeling approaches have not been clinically reliable^14^. We seek to build on prior attempts to predict later language outcome status from earlier MBCDI vocabulary assessment by incorporating recent advances in machine learning and network science.

### Graph-theoretic measures of lexico-semantic structure in expressive vocabulary

Network scientists have built on mathematical advances in graph theory to gain insights into basic mechanisms in cognition and learning—including factors that drive early word learning. For example, in cognitive neuroscience, network science measures have characterized the complex dynamics of neural structure and function to illustrate how the brain flexibly organizes information flow among neural areas according to the sensory, cognitive and learning demands of a task^15^. Similarly, network science studies have illuminated patterns in growth of early vocabulary acquisition that illustrate how early vocabularies are organized in multiple dimensions.

This network science approach to early vocabulary modeling involves the development of a network (or graph), comprising a set of nodes (or vertices) that are connected via a set of links (or edges). Nodes connected by links are neighbors, and neighborhoods are built from larger supersets of nodes and neighbors^16^. Graph-theoretic (i.e. network) representations of early vocabulary include the words in a child’s expressive vocabulary as the nodes, with semantic connections among words denoted by links in the network. We define links in each child’s individual lexico-semantic network as from their MBCDI-reported vocabulary items that share overlapping semantic features, following prior work^17,18^. Importantly, using semantic features to describe relations among words in early expressive vocabulary seems to explain variability in lexical processing in toddlers^19–21^, and also explains unique variance in multiplex networks that model patterns in early word learning^22^.

Network graph representations of lexico-semantic structure in vocabulary yield a number of measures. Here, we include several network measures that vary among late-talking toddlers with early language delays^23^. These measures are: (1) Mean degree, which reflects the mean number of semantic connections between individual words in the child’s vocabulary, (2) Global Clustering Coefficient, which provides an index of overall network connectivity, and (3) Mean Path Length, which measures mean semantic distance between words. We additionally include two other network measure suggested by an anonymous reviewer, (4) Betweenness centrality and (5) Harmonic Centrality. We then use machine learning approaches to incorporate these network measures to predict future language outcomes.

### Predictive analytics using machine learning methods

Recent advances in computer science coupled with the increasing availability of large datasets (i.e. “Big Data”) have stimulated rapid development of sophisticated classifier algorithms that can identify diagnostic markers from complex datasets. These algorithms can identify meaningful relations among multiple variables and outcomes that are not typically captured by traditional linear models, including non-linear, radial, and discontinuous patterns. Among the most robust of these algorithms, which we use in this project, is Random Forest modeling (RFs)^25^. RFs have demonstrated wide utility in a variety of classification problems, outperforming over 100 other classifier algorithms in a variety of datasets and testing conditions^24^. One benefit of RF algorithms is that they develop highly accurate classification solutions while requiring only minimal assumptions about the structure of the data, and are robust against over-fitting of selected training subsets. For instance, RF algorithms can develop accurate classifications of complex datasets comprising multiple feature types (e.g. binary, categorical, numerical), and distributions (i.e. normality is neither assumed nor required). Therefore, RF models require minimal pre-processing (e.g. no transformation or rescaling) to provide accurate and generalizable solutions.

RF approaches also support insights into which variables contribute most toward classification accuracy^25^. By comparing the accuracy of models with systematic inclusion or deletion of single variables (i.e. features), it is possible to assess the relative “importance” or degree to which an individual feature contributes towards classification accuracy. We apply RF approaches to our datasets to assess overall classification accuracy for later low language (LL) outcomes and to identify which variables support accurate classification.

### Our approach: using novel methods with big data to improve early identification of DLD

Though machine learning techniques are gaining popularity throughout clinical sciences for diagnostic classification with large biomedical datasets^26^, this paper represents the first attempt to apply these methods to predict future language outcomes. Similarly, though prior research suggests that graph-theoretic measures of semantic structure correlate with concurrent language delay^23^, other research does not find this same relation^27^. One potential unifying explanation could be that the importance of these factors changes across age and may relate to potential for longer-term delays. No group has yet explored how these measures predict long-term outcomes. Advances in this area have been hindered by the high cost of collecting large longitudinal datasets and the intensive computational requirements of graph-theoretic and machine learning analyses.

We overcome these challenges and capitalize on advances in multiple disciplines by analyzing large datasets collected by the first and second authors. Full description of these datasets—the Early Identification of Risk for Language Impairment (EIRLI) and Language Acquisition and Semantic Relations (LASER) datasets—will be detailed in the methods. By analyzing these individual and harmonized datasets, we address the following questions:

1. Which variables support prediction of later language outcomes? Prior attempts have identified promising variables including: demographic measures, vocabulary size, early word combinations and morpho-syntax^14^. We include these measures, while adding new measures of semantic structure in vocabulary. To determine which variables best support subsequent identification of LL outcomes, we assessed feature importance on models trained using all variables. Feature importance reflects each variable’s improvement of classification accuracy in the model relative to a model without that variable. Based on prior studies which highlighted differences in semantic structure in late-talking toddlers^23^, we expected that semantic structure measures should rank higher in importance than vocabulary size.
2. Does feature importance change with age? Based on prior analyses by Thal and colleagues (2013)^14^, we expected that demographic variables would better predict outcomes at younger ages, while MBCDI-derived measures would have greater predictive validity for older toddlers.
3. Is it possible to combine datasets to develop a general solution for diagnostic prediction ranging from preschool to school-age outcomes? We explored these data aggregation question in two ways: first, by assessing the diagnostic performance of models trained on an aggregated dataset (in supplemental analyses); and by testing whether models trained on one dataset generalize to the other.

## Results

### Initial evaluation of feature importance across datasets

The goal of these analyses is to identify variables that most strongly predict language outcomes across four datasets: EIRLI-younger (303 TD, 11 LL); EIRLI-older (374 TD, 16, LL); LASER-younger (73 TD, 12 LL); LASER-older (73 TD, 12 LL), using a selected set of 14 features that include network measures of semantic structure in each child’s early productive lexicon, overall vocabulary skill, grammatical ability, and demographic measures (see detailed description of all features in Methods). On each dataset, using a nested cross validation approach, we trained 100 permutations of each random forest model with all variables to identify the mean importance across permutations of each feature (i.e. to identify the mean percentage change that each feature contributed to model classification accuracy). Within each cross-validation fold, we initially carried out model training to select the top seven features ranked by importance for further model training and evaluation in the outer cross-validation loops.

#### Feature importance in younger datasets

**Figure 1:**
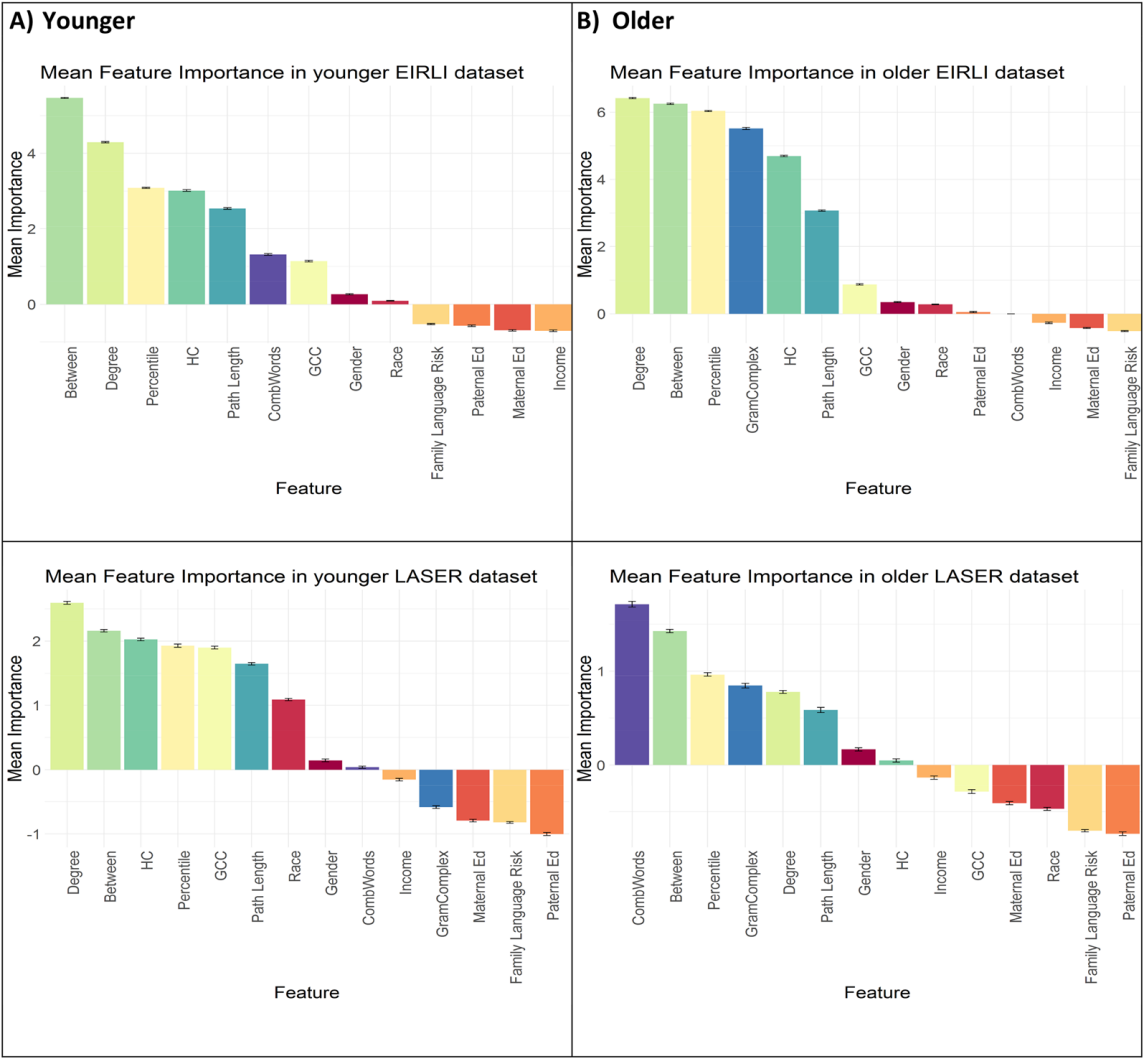
Feature importance rankings across younger and older datasets.

Figure 1a illustrates mean feature importance rankings across the younger datasets modeled with all feature data. Although there is some variability in the ordering of features across datasets, the MBCDI-derived features of semantic structure (including betweenness, degree, harmonic centrality, path length and GCC) and vocabulary percentile appear consistently in the top seven ranked features across both datasets.

#### Feature importance in older datasets

Figure 1b illustrates mean feature importance rankings across the older datasets modeled with all feature data. High-ranking variables reflect morpho-syntactic complexity of speech (combining words, grammatical complexity) and vocabulary size and structure (betweenness, percentile, degree, path length).

### Evaluation of model classification performance

#### Internal validation through repeated cross-validation in EIRLI and LASER datasets

Table 1 shows the binary classification performance of LL outcome from pruned models including the top seven features identified in the previous section for each model. Data reported in each column indicates model performance evaluation measures of: balanced accuracy (BalAcc); Sensitivity (Sens); Specificity (Spec); positive predictive value (PPV); negative predictive values (NPV); positive likelihood ratio (LR+); and negative likelihood ratio (LR−). All metrics illustrate moderate to strong classification performance across the board, with the relatively “weakest” performing dataset being the younger EIRLI data, though even in this case, the prediction classification performance is relatively strong.

**Table 1:** Model performance on each dataset when trained and tested using repeated-cross validation.

Train/Test	BalAcc	Sens	Spec	PPV	NPV	LR+	LR−
EIRLI-older	.91***	.99	.82	.19	1	5.50	0.01
LASER-older	.94***	1	.88	.61	1	8.33	0.00
EIRLI-young	.88***	1	.76	.13	1	4.17	0.00
LASER-young	.93***	1	.87	.58	1	7.69	0.00
Aggregated-older	.92***	.99	.85	.29	1	6.60	0.01
Aggregated-young	.92***	1	.84	.28	1	6.25	0.00

***Balanced accuracy significantly exceeds baseline balanced accuracy of .50 where all cases are classified as normal, all *p* values < .0001.

#### External testing of EIRLI and LASER model generalization to each other

These analyses represent the most challenging tests, as they explore whether models trained on either the EIRLI or LASER datasets can adequately classify outcomes in the alternative dataset. This test is particularly difficult because outcome variables across datasets were measured differently (EIRLI via self-report, LASER via direct testing) and at different ages (EIRLI at school age, LASER at age 3). Table 2 illustrates theses analyses, which show more modest performance than internal model tests. All models show relatively strong specificity and NPV, indicating that models tend to correctly identify children who do not have low language status compared to those who do.

**Table 2:** Model performance for external validation, training and testing on separate datasets.

Train	Test	BalAcc	Sens	Spec	PPV	NPV	LR+	LR−
EIRLI-older	LASER-older	.54***	.37	.72	.19	.87	1.32	0.88
LASER-older	EIRLI-older	.55***	.23	.87	.085	.96	1.77	0.89
EIRLI-younger	LASER-younger	.46	.15	.76	.098	.84	0.63	1.12
LASER-younger	EIRLI-younger	.50	.41	.58	.10	.92	0.98	1.02

## Discussion

Efforts to improve early identification of language disorders have the potential to positively impact the biomedical, psychosocial, educational and economic well-being of millions of children. Accurate and stable identification of language disorders from toddlerhood has been historically difficult, with prior efforts showing fair performance and requiring data collated on the same children from multiple ages^14^. In this study, we embark on an initial foray to ask whether we might improve identification accuracy from parental assessment of language skills at a single age by leveraging advances in classification via machine learning coupled with our own theoretically-informed network metrics of semantic structure in early vocabulary. The results are a promising initial step that suggests such approaches hold potential to improve diagnostic accuracy of early language disorder and delay. This project was guided by several primary questions. We interpret our study outcomes in light of these questions below.

Our first question sought to address which variables provide the best predictive performance for later language disorder and delay status. Here, we were primarily interested in whether measures of lexico-semantic structure in the early expressive lexicon might contribute additional predictive performance. Prior predictive modeling had only explored linear relations between simple vocabulary size and grammatical complexity in relation to later language risk. The present findings indicate that the semantic structure measures boost model accuracy, with these structure measures consistently falling within the top-ranked features that were retained in final models. Betweenness, Degree and Path length were retained in all models, while GCC and HC were retained in three out of four models. In fact, Betweenness and Degree were the top-two ranked features in three out of four models, suggesting that these two variables contribute more to model classification accuracy than other variables. Additionally, in all four models, multiple semantic structure measures ranked more highly than simple vocabulary size alone. We also included measures of morpho-syntactic (grammatical) complexity in datasets with older toddlers, and the presence of word combinations in datasets with younger toddlers. Both measures tap into grammatical complexity and ranked highly in several models, suggesting that these skills also contribute to classification accuracy and prediction of outcomes. In general, demographic variables tended to show somewhat lower variable importance ranking across models; although gender and race each contributed towards some of the top-ranked features, though less consistently than the MBCDI-derived measures of language skills. Demographic variables of family language risk, income, maternal and paternal education tended to contribute the least toward classification accuracy. These insights contrast with a number of other studies which point to the importance of socioeconomic factors in early language experience and growth^28–30^ as well as concentration of developmental language disorders and delays among families^31–33^. This difference between the widely noted impact of SES on language skills and our findings might be explained by a recent meta-analyses of numerous risk factors in DLD which suggested that while socioeconomic measures and family history are *statistically* significant predictors of language outcomes, they did not meet a bar for *clinical* significance as predictors of language disorders^34^, which we also seek to infer via markers of classification accuracy in the current project. We should note, however, that both of the currently modeled datasets under-sample lower-SES populations, compared to the true demographic distribution in the United States. Therefore, it is possible that this sparsity reduced the potential of these variables to contribute predictive power in these samples. Nevertheless, the fact that other demographic variables do contribute towards classification accuracy is consistent with current recommendations for pediatric practice which incorporate demographic indices of risk into developmental surveillance^35^. At the same time, the strong performance of other grammatical and lexico-semantic factors suggests current screening practices might be improved with the inclusion of grammatical and semantic structure assessments of early language skills. From a theoretical perspective, the robust rankings of MBCDI-derived measures suggest that the roots of language disorder are likely to develop from differences in learning aspects of semantic and morpho-syntactic structure in language.

Our final questions sought to evaluate model accuracy in early identification of low language outcomes. These questions have primary importance to clinicians who wish to identify children who stand to benefit most from early language intervention before preschool entry. Armed with several “rule-of-thumb” thresholds and prior linear modeling outcomes as our guide, we can assess the models’ overall ability to identify language delays and disorders in toddlerhood.

Model accuracy was highest when tested on untrained data drawn from a model’s own dataset distribution via repeated cross-validation (Table 1). These models show overall excellent sensitivity and specificity (ranging from 0.82 to 1), and moderate to excellent positive and negative likelihood ratios. Balanced accuracy (ranging from 0.88 to 0.94) always significantly exceeded 0.50, which would result in cases where models simply categorized all children as falling within the TD category (as may often happen when parents are advised to “wait and see” to delay identification). Crucially, these models show classification improvement when compared to prior linear modeling with the EIRLI dataset (where mean sensitivity, specificity, and balanced accuracy was 0.54, 0.84, and 0.70, respectively)^14^. While further work is needed to replicate and validate our findings, this improvement attests to a promising potential to develop clinically useful models that build on large datasets to predict preschool and school aged language outcomes from toddlerhood.

Models performed less well when predicting cases in the alternative dataset (Table 2). These cross-dataset predictions represent the most difficult assessment in our study. Several aspects of our modeling approach were geared towards reducing potential overfitting and promoting model generalizability, such as choice of algorithm and feature pruning practices. Nevertheless, we found that models trained on either the EIRLI or LASER dataset alone were relatively poorer at predicting cases from the alternative dataset.

There are several reasonable explanations for this decrement in cross-dataset performance. First, LL status was defined differently between datasets. In the EIRLI dataset, LL status was assessed by whether a language or reading disorder had been identified in school between ages 4–7—an assessment dependent on initial parental report of positive identification in school (though later verified by the researchers). In contrast, the LASER project screened all children at age 3 for presence of a language delay using a single standardized measure of CELF-P2. These assessment approaches may have differentially tapped into endophenotype subgroups of DLD that show relatively more pronounced difficulty in phonological short term memory (STM) vs. morpho-syntactic skill^36^. Specifically, the phonological STM endophenotype is often missed in younger children but emerges in reading difficulties in word decoding at school age, while the morpho-syntactic endophenotype is more easily captured at younger ages. Future work is needed to explore how endophenotype distributions affect early identification by capturing more detailed outcome measures that distinguish between these groups.

Our groups were also assessed at different ages, and epidemiological studies suggest that prevalence of LL is likely to be higher than typical identification in school settings. For example, in a sample of 216 children with DLD who had been identified in an epidemiological study, only 29% of parents had been informed of their child’s speech or language problem^1^. Because the EIRLI study relied on parental report of identification in school, rather than fully screening the sample, the EIRLI dataset likely under-estimated the number of LL cases. A second difference is that each dataset measured outcomes at different ages (LASER at 36–37 months; EIRLI at 4–7 years). In this case, the LASER dataset might over-identify delays in preschool that resolve before elementary school. These two factors (screening method and age) are also likely to account for the differences in LL prevalence among datasets (LASER: 14%, EIRLI: 4.3%). Dataset distribution comparisons (in Supplemental Material) also illustrate differences in the distribution of demographic variables, potentially reflecting geographical differences (LASER was collected in a medium-sized city in northern Florida, whereas EIRLI is drawn from a large metropolitan area in southern California). These sampling differences speak to a need for future work with geographically- and demographically-diversified samples.

An additional limitation stems from the dataset sample size. Although the studied samples are relatively large compared to many empirical studies exploring DLD, there was a relatively small number of positive cases overall. While we incorporated synthetic data sampling approaches (SMOTE) to generate robust models, future work is needed to capture larger numbers of positive cases. Fortunately, this goal is readily achievable. The MBCDI is widely adapted and easily administered, with digital formats that allow for web and mobile-device enabled versions that can be automatically scored. This instrument has already been deployed in large-scale projects with more than 1000 children in several regions^37,38^. The rich set of words in the MBCDI supports comprehensive network modeling of early vocabulary growth in toddlers, which in turn, supports rich insights into the nature of early vocabulary growth and delay. However, the lengthy nature of these parental report vocabulary checklists contrasts with other early screening measures, which prioritize brevity. For example, another common early screening measure, the Ages and Stages Questionnaire, includes many fewer questions that tap into early language skills. A promising next-step forward is to develop collaborative projects that merge rich early-screening datasets from multiple measures with data on school-aged outcomes to optimize the ability to create effective and efficient measures of early language risk.

With recent advances in clinical, behavioral, and computational fields, developmental scientists stand on the precipice of a new era of early detection advancements that promise to improve the lives of millions. We advance this cause in an initial attempt to apply machine-learning approaches to theoretically-derived measures of language skills to demonstrate that these new tools have the potential to yield practical improvements in early detection of language delay and disorder.

## Methods

We use two large, longitudinal datasets that include MBCDI-derived measures of early vocabulary skills and demographic variables along with outcome measures of later language/reading delays. Below, we describe each dataset before outlining the pre-processing and analytic plans.

### EIRLI dataset

The Early Identification of Risk for Language Impairment (EIRLI) dataset is part of a larger project developed by the second author to explore how early gesture, vocabulary and language skills unfold in early childhood (see Thal et al., 2013 for a detailed description of this project). In addition to collecting demographic information and MBCDI data, researchers followed up with the families at school age (once a year, between 4 and 7 years of age) and asked parents to report any diagnosis of a language or reading disorder. Researchers verified these reports via documentation from school records or a clinician. Language/Reading disorder outcome status was marked as “positive” if parents reported and verified any language or reading issue at any of these ages. Our analyses focus on the subset of this sample for which item-coded MBCDI data exist at 16 and/or 28 months (391 total), of which 17 (4.3%) later reported a school-age language/reading disorder.

### LASER dataset

The Language Acquisition and Semantic Relations (LASER) archive stems from the first author’s project designed to evaluate how MBCDI-derived semantic structure measures relate to early language development. Children in this dataset were followed between 18 and 36 months. Parents completed the Words and Sentences form of the MBCDI at quarterly intervals from 18 to 30 months, and children were evaluated for language delay at 36 months using the Clinical Evaluation of Language Fundamentals—Preschool 2 (CELF-P2^39^). We selected demographic and vocabulary variables from the LASER dataset that would align with those of the EIRLI dataset, including vocabulary data at 18 months, and 27 months, which were time points nearest to available EIRLI dataset time points of 16 and 28 months. We also selected the same demographic variables as available in the EIRLI dataset. The total dataset includes 85 children, of which 12 (14%) qualified for language delay categorization based on sensitivity and specificity cutoff scores on the CELF-P2 (standard score < 85) at 36 months.

Additional information about the distribution of language skills and demographics in the EIRLI and LASER samples are described in supplementary material sections (supplementary Table 1, and supplementary Figs. 1–5). These analyses highlight patterns in data missingness across samples (supplementary Fig. 1), and some differences in the distribution of variables, (supplementary Fig. 2). There are also patterns of correlations among variables (supplementary Fig. 3). However, concerns about multi-collinearity are mitigated by the use of RF approaches in modeling combined with feature selection and pruning which are robust against multi-collinearity^40^.

There are several notable differences between the datasets. First, there are geographic differences: EIRLI was collected in a large metropolitan region in southern California, while LASER was collected in a medium-sized city in northern Florida. The language outcome measures and ages in the EIRLI and LASER dataset are not identical. While the 3-year-old language delay CELF-P2 measures in LASER indicates an elevated risk of persistent delay, it is not a definitive language disorder, whereas the criteria in the EIRLI dataset represent a definitive language disorder diagnosis. Therefore, we assign an umbrella term—Low Language (LL) Outcome—to refer children in both groups who were either language delayed or disordered based on the criteria specified in each dataset.

### Ethical considerations

Parents in both datasets initially provided informed consent to participate in these projects which both sought to explore how early language skills relate to later language outcomes. Both projects received institutional review from the San Diego State University Institutional Review Board (EIRLI) and the Florida State University Institutional Review Board (LASER) and all methods were carried out in accordance with relevant guidelines and regulations. Our analyses involve secondary analysis of de-identified data subsets from both projects.

### MBCDI derived measures

We incorporate several measures from the MBCDI in these analyses including measures of vocabulary size, structure, word combinations and grammatical complexity.

#### Vocabulary size

Due to the differences in age across datasets, we include percentile measures based on normative sampling for the MBCDI^11^, instead of raw scores. Percentile measures provide consistent comparisons across datasets and age groups.

#### Vocabulary structure

We calculate several measures of semantic structure in MBCDI-reported vocabularies using graph-theoretic modeling of individual toddlers’ noun-feature networks. First, networks are constructed by assessing which of each child’s MBCDI-produced nouns share semantic features using a recently developed dataset of semantic feature norms for all noun items within the MBCDI^18,19^. Nodes in each network are represented as nouns in the toddler’s expressive vocabulary, and links are represented by nouns that share at least two semantic features in common. Based on these noun-feature networks, we calculated three connectivity measures that have been previously identified as varying between early-talker and late-talker vocabularies^23^: (1) Mean Path Length (MPL), (2) Global clustering coefficient (GCC), and (3) Mean Degree (MD), and two additional measures suggested by a peer reviewer (4)Betweenness Centrality (BC), and (5) Harmonic Centrality (HC)^41^.

#### Description of network measures

**Figure 2:**
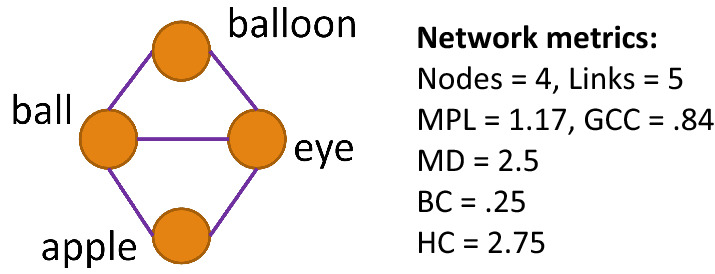
An example noun-feature network with four nodes (words) and five links (indicating multiple overlapping semantic features between words.)

We incorporate five network connectivity measures into our analyses: Mean Path Length, Global Clustering Coefficient, Mean Degree, Betweenness Centrality, and Harmonic Centrality. Figure 2 illustrates these measures in a small noun-feature network, and detailed description of each measure follows.

1. *Mean Path Length (MPL)*. Path length (L) is defined as the shortest number of “hops” between any pair of word nodes via semantically-overlapping feature links, with mean path length as the mean distance between all connected node pairs in a network. For example the path length between *apple* and *balloon* in Fig. 2 is 2 (L_apple,balloon_ = 2), and the mean path length of all word pairs in the network is 1.17 (MPL = 1.17). Networks with shorter mean path lengths have more efficient information transfer properties^42^. In the context of lexical development, shorter mean path length is likely to support efficient activation and retrieval of word meanings.
2. *Global clustering coefficient (GCC)*. Clustering coefficient (C) for any node is the total number of triangles between the node’s neighbors divided by the total possible triangles. In Fig. 2, the clustering coefficient of *eye* is 0.67 (C_eye_ = 0.67). GCC measures the overall connectivity of the nouns in each toddler’s vocabulary. GCC is calculated as the total number of words that are connected in closed triples divided by the total number of connected triples^43^. A connected triple is any set of three words (nodes) that shares semantic links (e.g. in Fig. 2, *apple/eye/balloon* form a connected triple), and closed triples include cases where all three nodes are interconnected (e.g. *eye/ball/balloon* form a closed triple in Fig. 2). The GCC of the noun-feature network in Fig. 2 is 0.83. GCC values range from 0 to 1; a 0 value denotes that the child’s lexicon has no connected triples, and 1 indicates that all triplets are closed. Toddlers with a higher GCC have higher semantic connectivity of their vocabulary, with a higher proportion of vocabulary in semantic word “neighborhoods” compared to children with a lower GCC.
3. *Mean Degree (MD)*. Degree (*k*) of each vocabulary item is measured by the number of other nodes for which there is shared feature overlap (e.g. the *k*_eye_ = 3 in Fig. 2). Mean degree (MD) is calculated as the average degree of all items in each toddler’s vocabulary network (MD in Fig. 2 = 2.5). Children with higher mean degree vocabularies know more words with direct semantic neighbors compared to children with lower mean degree vocabularies.
4. *Mean Betweenness Centrality (BC)*. Betweenness centrality of each vocabulary item is measured as the number of times that word appears on the shortest path to all other node pairs in the network. This measure represents the degree to which a word is central to the connections between other words in a network, by appearing “between” other word pairs. Mean betweenness centrality is calculated as the mean betweenness of each node across all words in the child’s network.
5. *Mean Harmonic Centrality (HC)*. Harmonic Centrality is a variant of closeness centrality, which are both measures that capture how closely nodes in a network connect to other nodes. Harmonic centrality is calculated as the inverse of the sum of all distances of a node between all other nodes, and then divided by the number of words minus 1. Values range between 0 (indicating a word is not connected to any other words) to 1 (a word is connected to every other word). This measure (unlike closeness centrality) can deal with cases where there are unconnected nodes in a network, as is often the case in early vocabulary development. Mean harmonic centrality is calculated as the average harmonic centrality of all words in each child’s network.

#### Combining words

One item on the MBCDI that is often used as a milestone marker of language delay is whether a child has begun to combine words into multi-word utterances^44^. This question is posed as a single item to parents, which states, “Has your child begun to combine words yet, such as “nother cracker” or “doggie bite?”. There are three response options: ‘Not Yet’, ‘Sometimes’, and ‘Often’, with ‘Not Yet’ scored as a 0, and ‘Sometimes’ and ‘Often’ as 1.

#### Grammatical complexity

The MBCDI also asks parents to indicate whether their child is producing a number of morpho-syntactic markers. Items ask caregivers to compare two example utterances that vary in morpho-syntactic complexity and then identify which examples best correspond to their child’s language (e.g. the first item asks parents to select between *two shoe* vs. *two shoes*). In a previous analysis of the EIRLI dataset^14^, this grammatical complexity measure at 28 months predicted unique variance in later outcome status. Therefore, we include this measure in our analyses.

### Analytic approach

The data analysis pipeline comprises initial data selection and merging, modeling using cross-validated datasets, selecting features within cross-validated folds, and evaluating model performance. Additional exploration and description of data distributions is described in supplementary analyses. Dataset merging, model training and evaluation steps follow below.

### Dataset merging and variable inclusion

We initially selected identical variables for model training across datasets to facilitate model comparison and evaluation. We included vocabulary size (as MBCDI percentile), vocabulary structure (MPL, GCC, MD, BC, HC), and demographic variables (parental education, household income, race, gender, family history of speech or language disorder). To further support model building and evaluation across both datasets, we aggregated the two datasets into a younger (EIRLI 16 month and LASER 18 month) dataset and older (EIRLI 28 month and LASER 27 month) dataset, with identical demographic and vocabulary size and structure variables. Although LL status was determined using different measures and ages between the two datasets, we nevertheless merged all positive and negative outcome results into a single column. This procedure allowed us to determine whether it would be possible to develop general classifier models that could identify risk for poor language outcomes across the preschool and early school-age period.

### Initial model training and feature ranking

We sought to identify which variables predict later outcomes across datasets using a random forest (RF) modeling approach. We were particularly interested in determining whether variable importance would change across aggregated older and younger datasets, and whether there is agreement between EIRLI and LASER datasets at each age. We provide a general description of RF modeling in Supplementary materials, and model parameters below.

#### Description of random forest modeling techniques

RF models are developed by building a collection of individual “weak learner” decision trees (i.e. each individual tree is prone to over-fitting and over-generalization). These decision trees are fit on random orderings and subsets of variables within the dataset, and generate “split points” at each tree which reflect binary (Yes/No) predictor thresholds (e.g. Is gender Male? Is vocabulary percentile between 25 and 75?). Each random subset of predictors is termed a “node” and predictor thresholds are selected to optimize model accuracy. This process is iteratively repeated to produce trees of varying “depths” with multiple nodes, resulting in a single decision tree. This process is repeated multiple times (500 in the current study), creating a “forest” of trees, which when combined results in a robust “stronger learner” model that is less susceptible to overfitting^25^. Importantly, as these models generate solutions using decision trees, rather than linear regression, they are able to accurately identify both linearly- and nonlinearly- separable solutions. Randomly selected subsets of the data and subsets of variables are repeatedly selected and subjected to a decision tree algorithm. This random variable selection process serves to combat issues with multi-collinearity in large datasets by reducing correlation among decision trees.

#### Random forest modeling parameters and feature ranking

To build initial models we use a nested cross-validation approach using a random forest algorithm, with three-fold repeated cross validation and 10 repetitions in the outer loop, five-fold repeated cross validation and 10 repetitions in the inner feature selection loop, using the caret package in R^45^. The goal of this nested approach is to reduce possibility of data leakage by carrying out feature selection on cross-validated subsets of data that do not contribute to the final tuned model^46^. Random forest models were set to a standard tree size (ntree = 500 trees), and tuned for optimal *mtry* (i.e. the number of features included at each split of each tree, ranging from 2 to the total number of features in each dataset). Due to the relatively low incidence of children with LL in all datasets, we employed Synthetic Minority Oversampling Technique (SMOTE) to balance modeling data^47^. This balancing method generates synthetic samples in training data using a k-nearest neighbors algorithm to generate new and synthetic samples of the minority (positive diagnostic) class with similar properties to the existing dataset. Model training was repeated 100 times on each dataset, and feature selection was carried out 100 times within each cross-validation fold and repeated 10 times.

Within the inner loop of the nested cross validation procedure, we evaluated which features best contribute to model accuracy using a mean model importance metric across all 100 training instances. Importance for a single model is calculated by comparing the difference in classification accuracy (i.e. the percentage of cases correctly classified) of the full model compared to a model with that single feature removed.

Mean feature importance was then ranked, and the top seven features were selected for final model training in the outer cross-validation loop. The goal of pruning features to a subset is to reduce potential for model overfitting, and thereby increase the potential of models to improve their potential for external generalizability. Because 14 total features are used in initial full model training, seven features represents a selection of the top half of variables.

### Model performance evaluation

In all cases, we evaluate model performance on untrained examples in the dataset. For internal validation, we ask how well models correctly predict cases drawn from their initially trained datasets by evaluating performance on data that are held back through repeated cross-validation (i.e. by testing on the “out-of-bag” samples). This internal validation approach assesses how well models predict language diagnostic status on cases that are drawn from their original dataset distribution (e.g. How well do models trained on the EIRLI dataset perform on other values in the EIRLI dataset?). During external validation, we evaluate how models trained on one dataset generalize to another (e.g. if models trained on EIRLI predict outcomes in LASER and vice versa). These kinds of external assessments represent the most difficult tests of model performance, and allow us to address questions about whether and how models trained to predict LL status at preschool age (LASER) vs. school-aged (EIRLI) transfer to each other.

**Figure 3:**
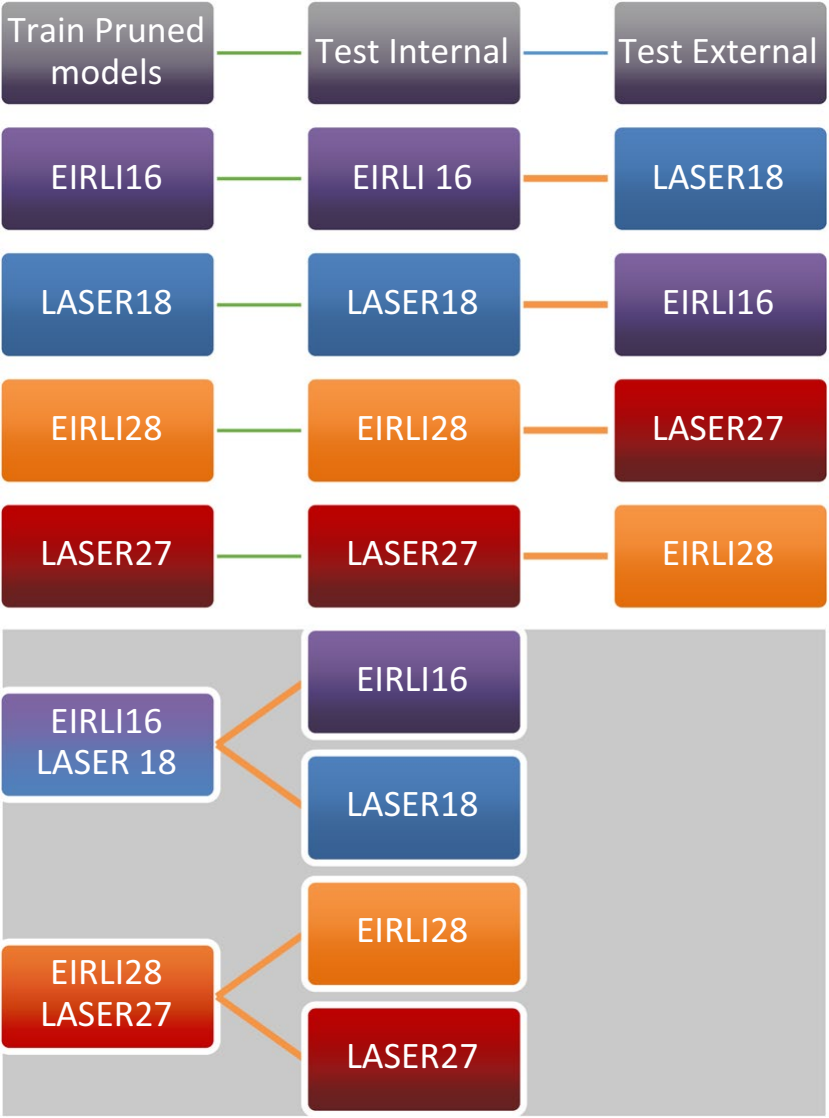
Visual schematic of strategy for model training and testing on internal and external cases from each dataset. Aggregated dataset models (depicted on gray background) reported in Supplemental Material.

Figure 3 outlines a visual schematic of our strategy for training and internal and external model performance testing across datasets.

#### Classification measures

During the outer cross-validation model training loop, we assess the performance of each model permutation trained via repeated cross-validation by assessing the accuracy of classification on untrained cases. These results are collated into a confusion matrix, which is a 2 × 2 table that represents correct vs. predicted classification of LL status^48^. We use confusion matrices of model performance to calculate several metrics including: balanced accuracy; sensitivity; specificity; positive and negative predictive value; and positive and negative likelihood ratios^49^.

We use confusion matrices of model performance to calculate several metrics including: balanced accuracy; sensitivity; specificity; positive and negative predictive value; and positive and negative likelihood ratios. Sensitivity is defined as the proportion of children who are classified by the models with positive LL status out of all of the children with true LL, while specificity indicates the proportion of children that are classified as not having LL out of those who truly do not. Balanced accuracy is calculated by taking the mean of sensitivity and specificity values. This measure provides an overall index of model performance while balancing for differences in positive and negative LL class size. Positive predictive values (PPV) indicate the proportion of times the model correctly indicates the presence of LL out of all instances where LL is present, while negative predictive values (NPV) indicate how often the model correctly indicates the absence of LL out of all instances where it indicates LL is not present. All model evaluation metrics, except for likelihood ratios, vary between 0 and 1 with higher values indicating better performance. Plante and Vance (1994) recommended that language identification assessments with sensitivity and specificity ranging between 0.9 and 1 are considered “good,” while measures ranging between 0.8 and 0.89 are considered “fair.”^50^ Though these thresholds are helpful in establishing a rule-of-thumb metric for identification quality, we are additionally interested in assessing how current modeling approaches improve on prior classification attempts using MBCDI derived measures. In prior work using linear modeling approaches to classify outcomes with the EIRLI dataset^14^, mean model sensitivity was 0.54 (range = 0.41–0.69); mean specificity was 0.85 (range = 0.69–0.95); mean balanced accuracy was 0.70 (range = 0.68–0.71); mean PPV was 0.26 (range = 0.14–0.37); and, mean NPV was 0.96 (range = 0.95–0.97). Although there are important differences between this prior work and the present project, we take these measures as a reasonable “baseline” by which to compare performance in the current study. We additionally calculate positive and negative likelihood ratios (LR+ and LR−, respectively) for model performance following recommendations for appraising clinical evidence in communication disorders when clinical prevalence is relatively rare^51^. LR+ ranges from 1 to infinity, and higher values reflect greater confidence that a positive test classification indicates a true positive result; LR+ values above 3 are considered to be moderately positive, and 10 is very positive. LR− ranges from 1 to 0 and smaller values reflect greater confidence that a negative classification indicates a negative result; values below 0.3 are considered moderately negative, and below 0.1 are strongly negative. LR+ is calculated as: sensitivity / (1-specificity) and LR- is calculated as: (1 − sensitivity)/specificity. Although likelihood ratios are not reported in Thal and colleagues (2013), we calculated each of these measures based on their reported sensitivity and specificity. In that prior study, mean LR+ was 5.0 (range = 2.2–8.2) and mean LR− was 0.53 (range = 0.44–0.62), and we consider these likelihood ratios as an additional baseline comparison for the current project. Finally, in other pilot testing of the modeling approach using randomized “shuffled” labels, these models tended to converge on classification solutions that categorized all cases in the “normal language” or negative diagnostic category, yielding balanced accuracy of 0.5. Therefore, we conduct t-test comparisons of the balanced accuracy measures of trained models against 0.5 to determine whether our model performance exceeded this additional “baseline” threshold.

## Supplementary Information

Supplementary Information.

## Data Availability

Upon publication, we will publish the participant demographic and MBCDI-derived variables that contributed to these analyses at http://www.osf.io/uf3vp/.
