# Supplementary material for: Moving towards accurate and early prediction of language delay with network science and machine learning approaches
Source: Sci Rep. 2021 Apr 14;11:8136. doi: 10.1038/s41598-021-85982-0 (PMC8047042; doi:10.1038/s41598-021-85982-0)
Supplement: Supplementary file 1 — Supplementary Information. [file 41598_2021_85982_MOESM1_ESM.pdf]

Supplemental Material for

Moving towards accurate and early prediction of language delay with network science and machine learning approaches

Arielle Borovsky[1], Donna Thal[2], Laurence B. Leonard[1]

1- Purdue University, Department of Speech, Language, and Hearing Sciences, West Lafayette, Indiana

2- San Diego State University, School of Speech, Language, and Hearing Sciences, San Diego, California

Exploratory analytics. Given the complexity of the datasets, we planned several exploratory analyses to highlight similarities and differences across datasets and diagnostic categories. The analyses include distribution of missing values, data distribution across datasets, correlation among variables, and relations of variable distributions to outcome status.   Table S1 below illustrates the distribution of cases across datasets by diagnostic category.

Table S1. Description of sample size across diagnostic category in EIRLI and LASER datasets

**Diagnostic Category**	**LASER 18 months**	**LASER 27 months**	**EIRLI 16 months**	**EIRLI 28 months**
Normal Language	73	73	303	374
Low Language	12	12	11	16

Missing values. Figure S1 indicates differences in completeness of data across sets. In the EIRLI dataset, missing cases largely comprise children who are missing a MBCDI at 16 months; whereas the missing data patterns are more varied among the LASER dataset. Due to the larger number of missing cases and variability in data missingness in the LASER set, we use a random forest approach to impute missing data (Liaw & Wiener, 2002). This approach builds a random forest model for each variable, which is then used to predict missing variables from the remaining variables. All subsequent analyses are carried out using this imputed dataset.

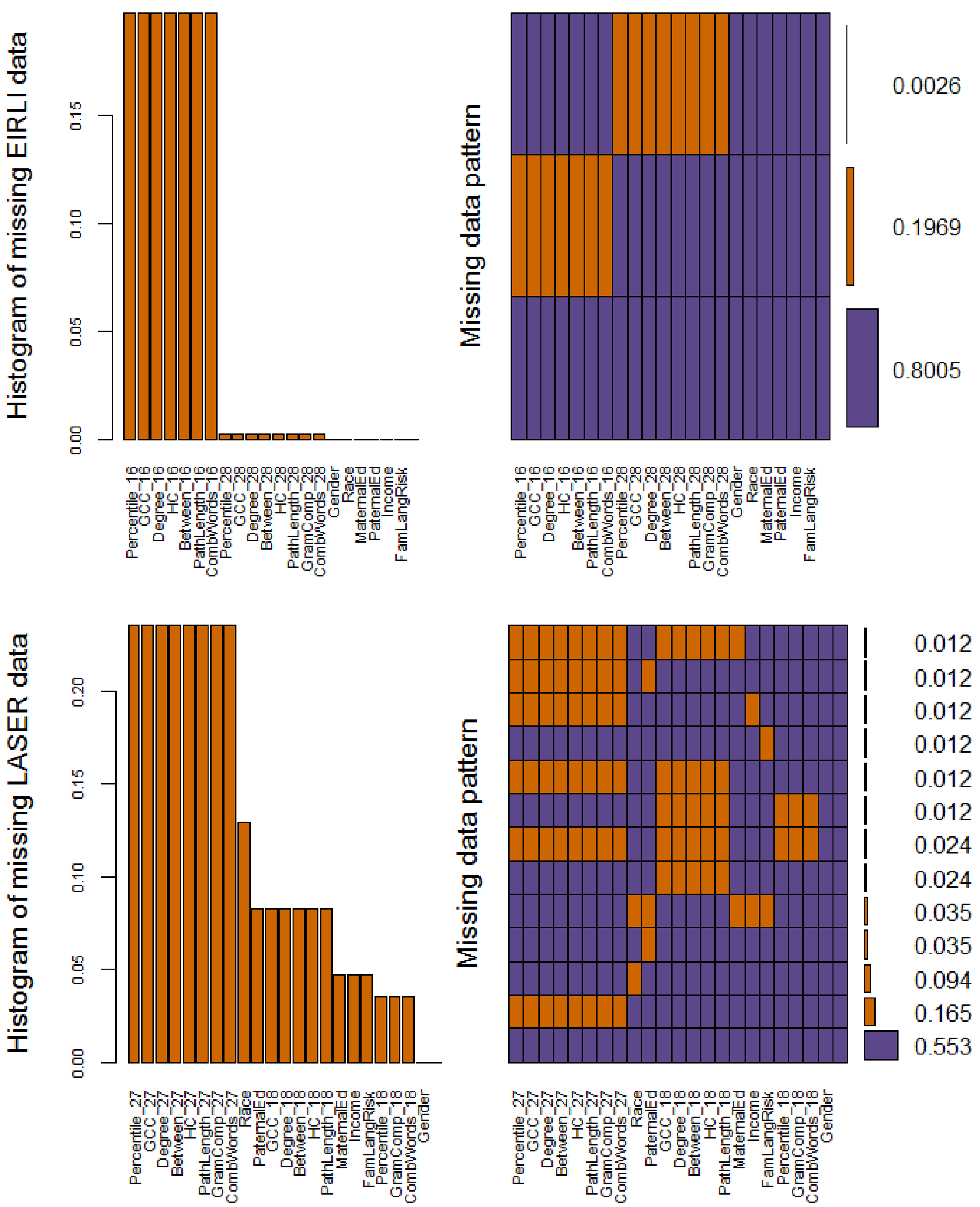

Figure S1. Missing data patterns across datasets. Missing data is highlighted in orange. Data indicate that the EIRLI dataset is relatively complete, and 80% of children have complete data across both time points, with missing data primarily driven by missing MBCDI data at 16 months. The LASER dataset has 55% complete cases, with missingness primarily driven by missing MBCDI data at 27 months (17.6%), and missing ethnicity information (9%).

Data distribution across datasets. Next, we compare whether the distribution of values across variables is similar across datasets. This comparison provides some indication of potential generalizability of model solutions across sets. We use two-sample Kolmogorov-Smirnov (KS) tests, a non-parametric statistical test that compares the shape of two data distributions (Goodman, 1954). Variables that exceed the FDR-corrected threshold for significance in KS tests indicate that the sample distributions for that variable are significantly different. We compare features across older and younger datasets in Figure S2. In the younger dataset, KS tests indicate that Income levels, likelihood of combing words are not distributed equally across EIRLI and LASER sets. In the older dataset, harmonic centrality, grammatical complexity, percentile, income, GCC, betweeness, degree, and combining words are not distributed equally between datasets.

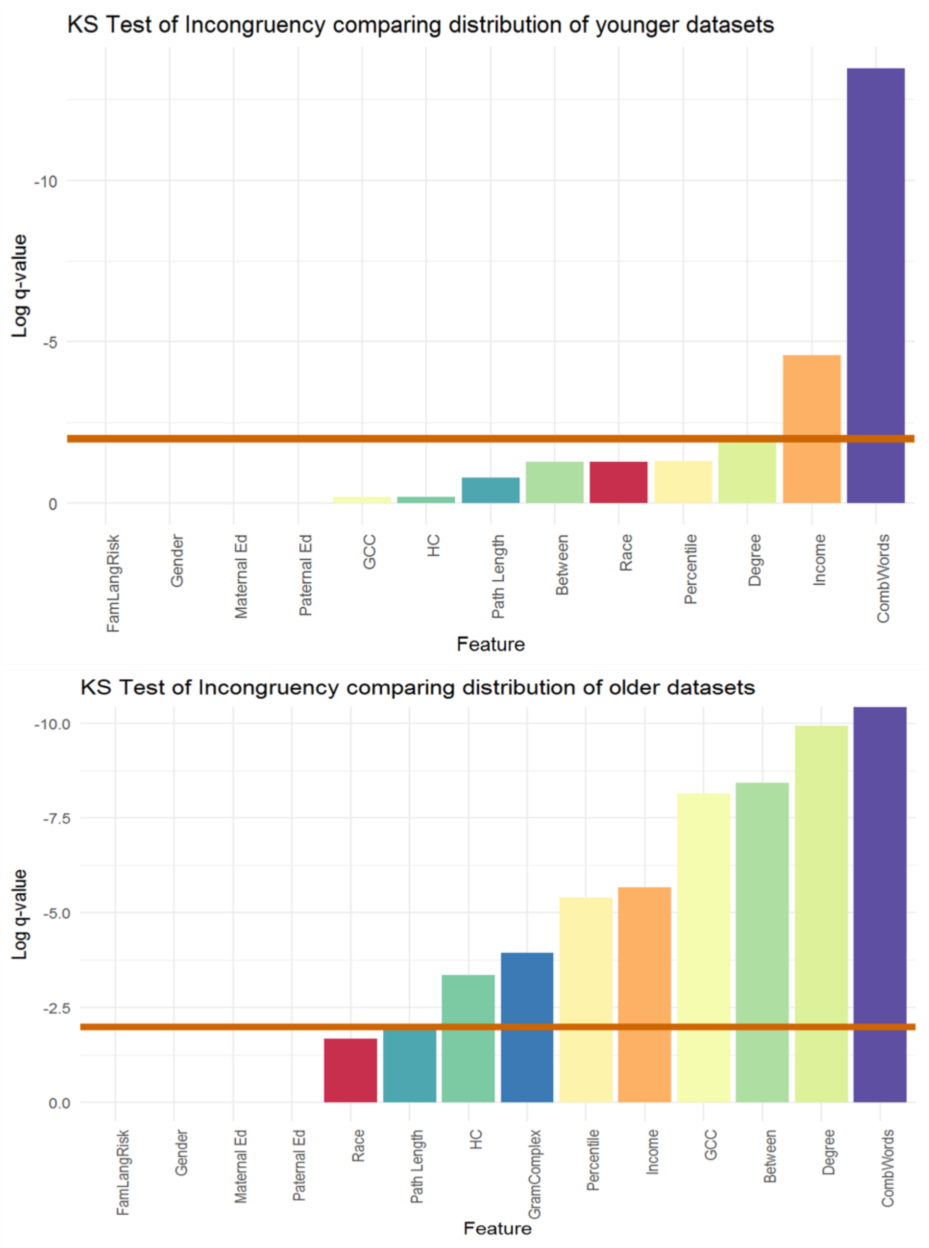

Figure S2. KS tests comparing data distributions in older and younger datasets. Orange line marks the log-alpha level of the FDR-corrected p-value, with threshold set to $p < .01$.

Pairwise correlations of features in each dataset. Figure S3 below illustrates pairwise correlation heatmaps in the EIRLI and LASER datasets. Both datasets illustrate robust correlations among MBCDI-derived measures of vocabulary size and structure, both within and across age. The relative lack of correlation between demographic variables and vocabulary suggest that each class of variable may contribute unique predictive power to models.

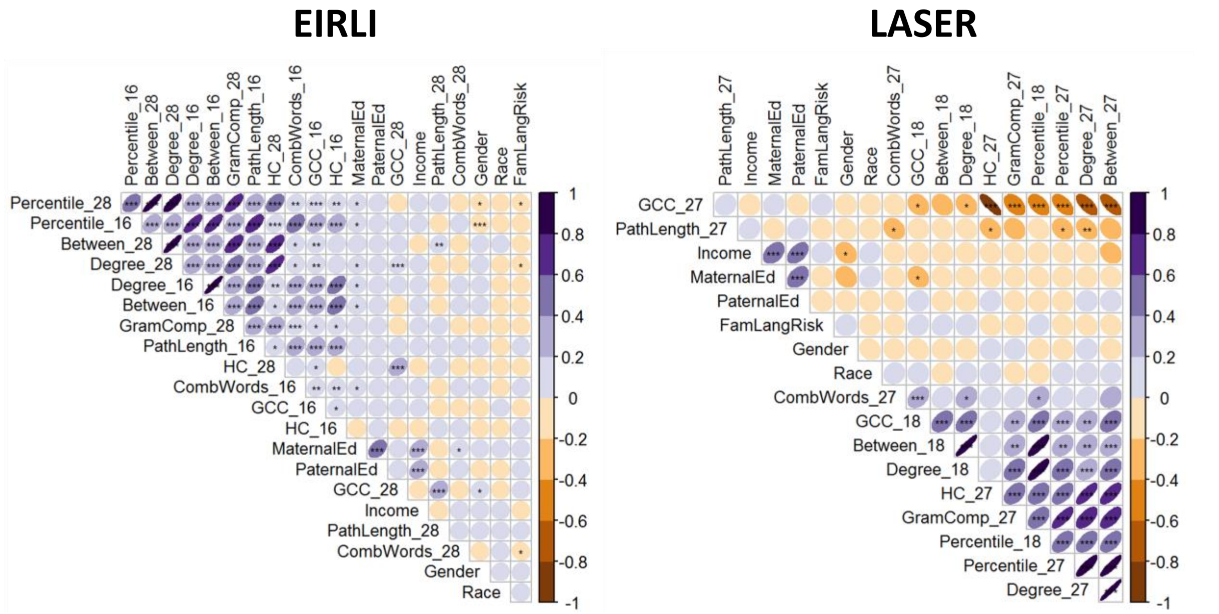

Figure S3. Pairwise correlations of all variables in each dataset, aggregated across age. Color scale and ellipses indicate size of correlation coefficients, and asterisks indicate significance thresholds, with * - p < .05, ** p< .01, *** p < .001

Data distribution by diagnostic category in EIRLI and LASER data. In Figure S4, we plot data means as a function of LL status across all datasets. These plots illustrate that, while most variables show some overlap in their mean distribution, some show relative differences as a function of LL outcome. These patterns are further explored in Figure S5, which illustrates the density of data distribution across cases with each LL outcome. Across both figures, we see cases where means do not vary in box plots, while there are notable differences in distribution in density plots. For example, Path Length in the younger EIRLI dataset does not vary in box plot means by LL outcome (Figure 4, "PathLength_16"), but shows clear distributional differences in the density plots (Figure 5). Although typical linear models cannot easily separate these kinds of distributional differences, machine learning methods, like random forest algorithms, are ideally suited for parcellating these non-linear relations between categories.

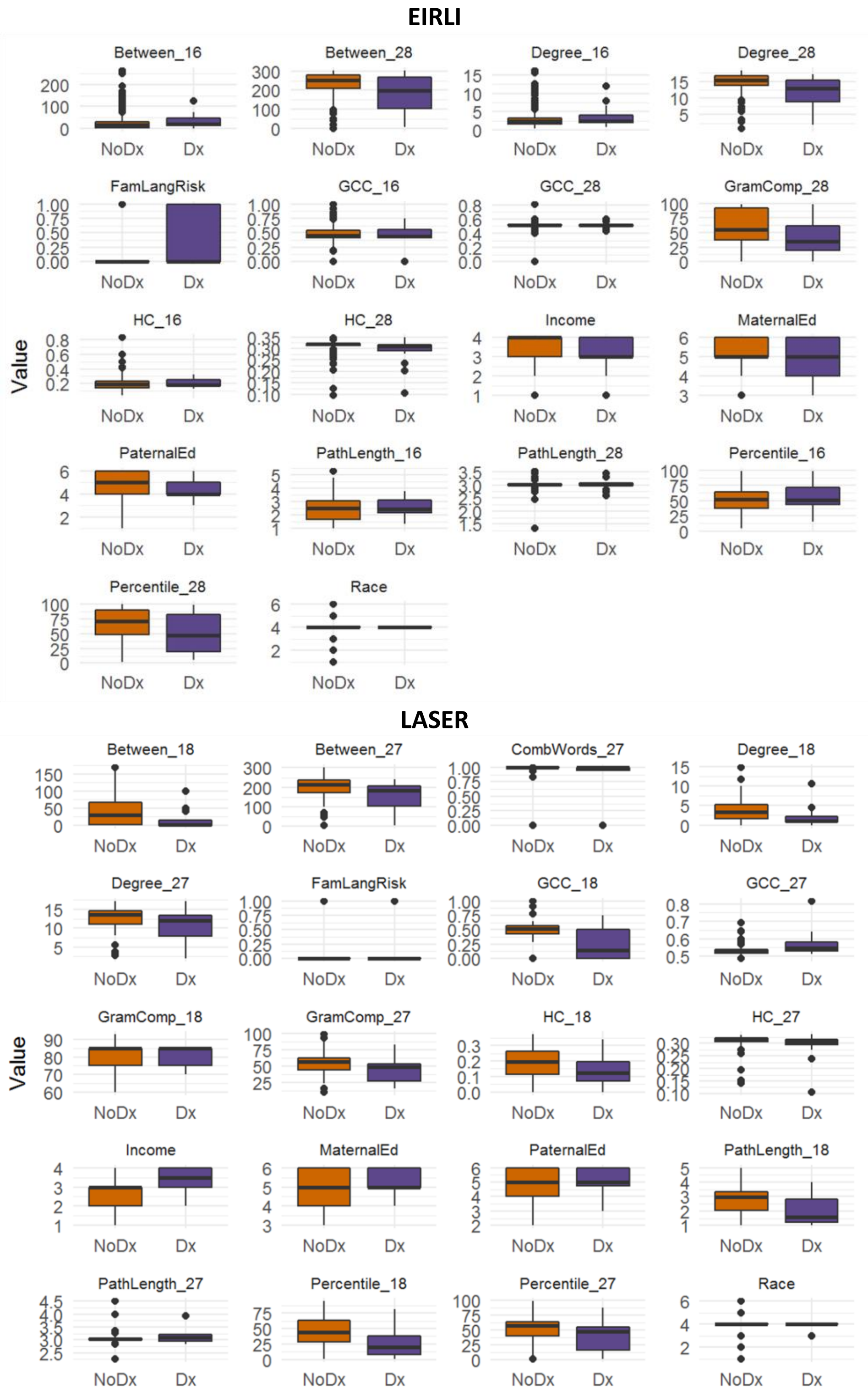

Figure S4. Box plots of data means as a function of LL status across datasets. Dx indicates positive cases with low language (LL) outcomes.

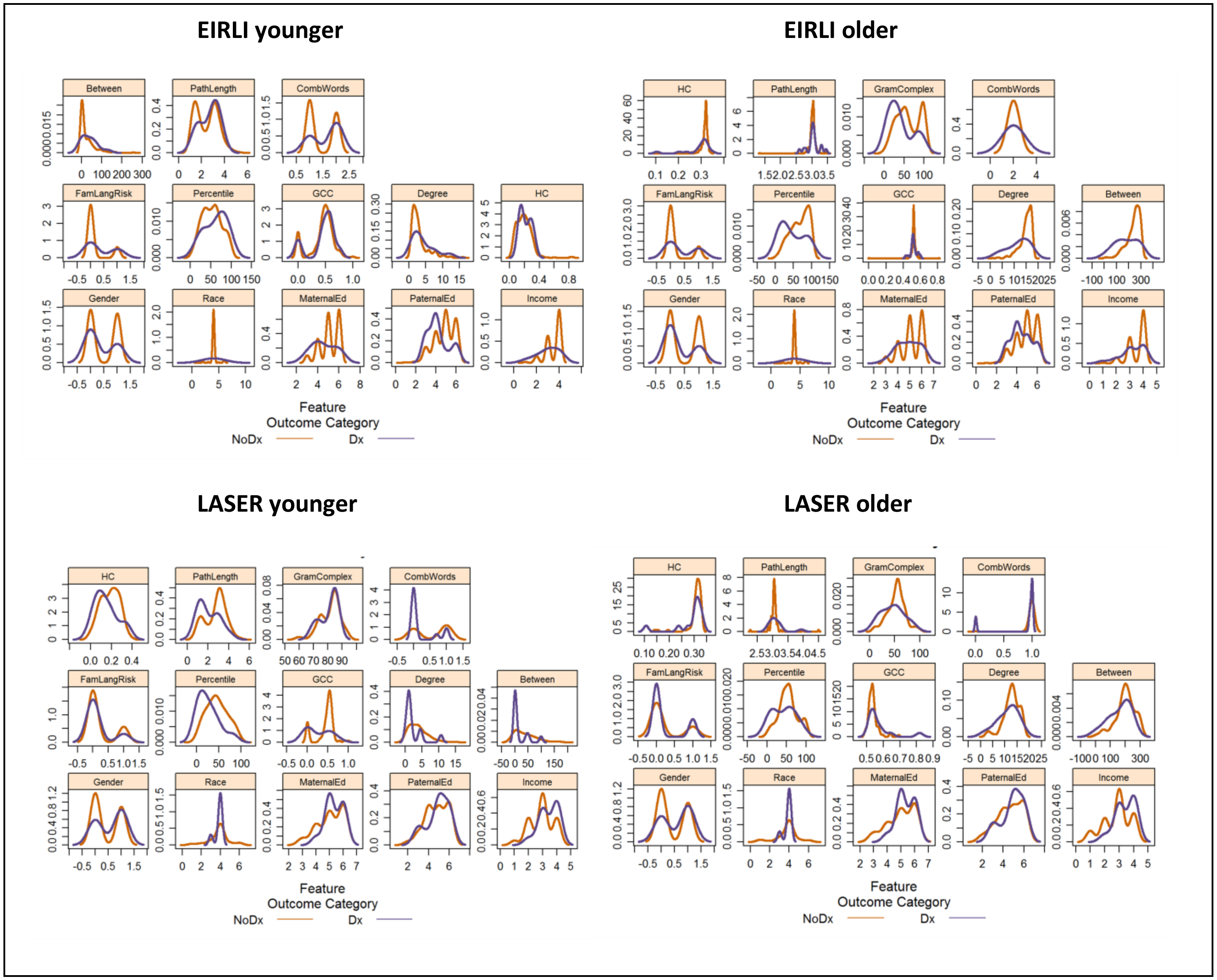

Figure S5. Density plots of feature distributions as a function of outcome across datasets.

Model training and testing of aggregated older and younger datasets

Feature importance across older and younger aggregated datasets. We aggregated data for younger (16-18 months) and older measures (27-28 months) in the EIRLI and LASER datasets to assess feature ranking and model classification performance. These results are illustrated below.

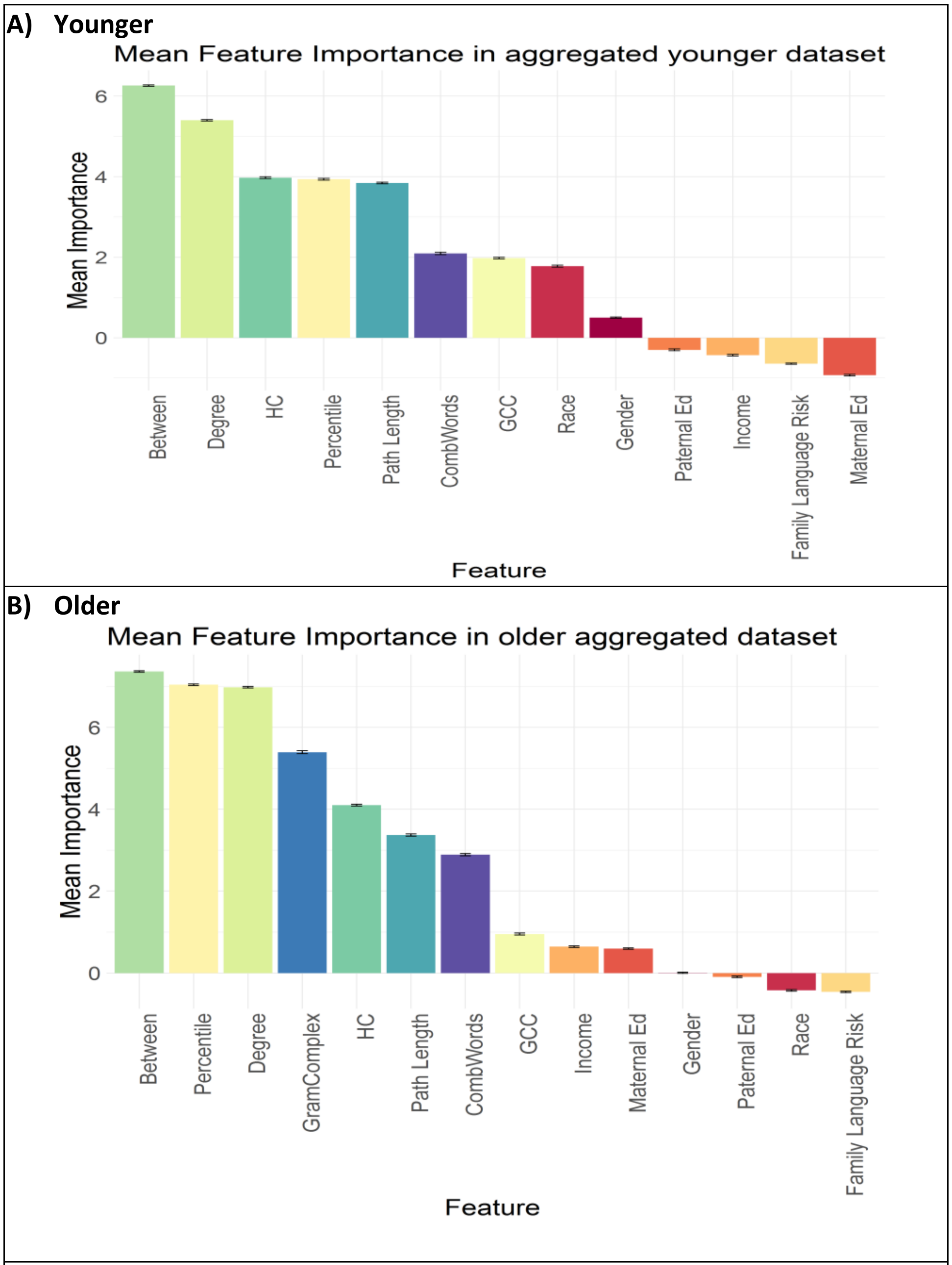

Figure S6. Feature importance rankings across aggregated younger and older datasets.

Internal validation of data subsets from older and younger aggregated data. The next analyses explore whether aggregated datasets would perform well on subsets of data from EIRLI and LASER datasets separately. Table S2 illustrates aggregated model performance when tested on subsets of data. Values generally indicate that models did a better job identifying outcomes in the EIRLI (vs. LASER) dataset. This difference in performance is likely due to the differences in training dataset sizes, with EIRLI including data from more children than the LASER dataset.

Table S2. Model performance for internal validation, training and testing on subsets of aggregated data.

Train	Test	BalAcc	Sens	Spec	PPV	NPV	LR+	LR-
**Aggregated - older**	**EIRLI - older**	.81***	.74	.89	.23	.99	6.73	0.29
**Aggregated - older**	**LASER - older**	.70***	.84	.56	.26	.96	1.91	0.29
**Aggregated - younger**	**EIRLI - younger**	.78***	.68	.87	.18	.98	5.23	0.37
**Aggregated - younger**	**LASER - younger**	.73***	.82	.63	.28	.96	2.22	0.29

*** indicates balanced accuracy significantly exceeds baseline balanced accuracy of .50 where all cases are classified as normal, all p-values < .0001

Model outcomes with an alternative random-forest approach: Extremely Randomized Trees (ExtraTrees).

A reviewer suggested we also inspect model performance when using extremely randomized trees, which is another random forest method that randomly generate tree node cut points, rather than seeking to create "optimal" splits in trees (Geurts, Ernst & Wehenkel, 2006). In Supplemental Tables S3 and S4 below, we report the results of these models, which are the analogous reports as main text Tables 1 and 2.

Table S3. Model performance on each dataset when trained and tested using repeated-cross validation using the ExtraTrees approach.

Train/Test	BalAcc	Sens	Spec	PPV	NPV	LR+	LR-
**EIRLI-older**	.93***	1	.85	.22	1	6.67	0.00
**LASER-older**	.96***	1	.92	.69	1	12.50	0.00
**EIRLI-young**	.89***	1	.79	.15	1	4.76	0.00
**LASER-young**	.94***	1	.87	.59	1	7.69	0.00
**Aggregated-older**	.93***	1	.86	.31	1	7.14	0.00
**Aggregated-young**	.93***	1	.86	.30	1	7.14	0.00

Table S4. Model performance for external validation, training and testing on separate datasets using the ExtraTrees approach.

Train	Test	BalAcc	Sens	Spec	PPV	NPV	LR+	LR-
**EIRLI-older**	**LASER-older**	.53	.49	.57	.20	.72	1.14	0.89
**LASER-older**	**EIRLI-older**	.49	.89	.10	.086	.63	0.99	1.10
**EIRLI-younger**	**LASER-younger**	.43	.11	.75	.078	.83	0.44	1.19
**LASER-younger**	**EIRLI-younger**	.47	.25	.68	.076	.90	0.78	1.10

Supplemental References

Geurts, P., Ernst, D. & Wehenkel, L. (2006). Extremely randomized trees. *Machine Learning, 63,* 3–42 https://doi.org/10.1007/s10994-006-6226-1
